# 

**Published:** 1812-04

**Authors:** 


### MONTHLY CATALOGUE OF MEDICAL BOOKS.

THE Physician's Vade-Mecum, containing the Symptoms, Causes, Diagnosis, Prognosis, and Treatment, of Diseases; accompanied by a select Collection of Formula, and a Glossary of Terms. By Robert Hooper, M.D. A new edition; crown 8vo. Murray.

Elements of the Science of Botany, as established by Linnæus, with 147 plates. Third edition; 8vo.—Bensley.

A Review of Mr. Everard Home's Practical Observations on the Diseases of the Prostate Gland, and of his important Anatomical Discovery. By Jesse Foot, Surgeon. 8vo.—Highley.

Plates of the Brain, represented in such a manner as to imitate Dissection. By Alexander Ramsay, M.D. 4to.—Edinburgh.

A new Edinburgh Dispensatory. By John Thompson, M.D. 8vo.—Edinburgh.

### *NOTICES TO CORRESPONDENTS.*

*We have not inserted the Letter signed L. N. because it appeared to us to contain some illiberal reflections on the practice and conduct of certain Practitioners in the Writer's immediate neighbourhood. Though some of his observations were just and sensible, others were unfit for public perusal, by disclosing particulars which are only meet for discussion amongst medical men in private. The respectability of the profession is not advanced by the avaricious principles, or ungentlemanly conduct, of some of its professors being obtruded on public notice.*

*We have been favored with communications from Doctor Denman; and Messrs. A. Fogo; E. L. Knowles; H.; S. L.; &c. &c.*

